# Early-Life Vitamin A Deficiency Induces Tissue-Specific Oxylipin Remodeling and Hepatic Inflammation

**DOI:** 10.3390/nu18121988

**Published:** 2026-06-19

**Authors:** Joseph Arballo, Jun Yang, Reina Engle-Stone, Kelly ZhaoZhao, Minghua Tang, Peng Ji

**Affiliations:** 1 Department of Nutrition, University of California, Davis, CA 95616, USA; jrarball@ncsu.edu (J.A.);; 2 Department of Entomology, University of California, Davis, CA 95616, USA; 3 Department of Food Science and Human Nutrition, Colorado State University, Fort Collins, CO 80523, USA

**Keywords:** vitamin A deficiency, oxylipin metabolism, liver fibrosis, inflammation, mouse model

## Abstract

**Background:** Retinoid signaling is implicated in regulating membrane-bound polyunsaturated fatty acids (PUFAs), which serve as substrates for oxylipin biosynthesis. Dysregulated vitamin A status and altered oxylipin profiles have both been associated with the development of metabolic diseases. However, whether early-life vitamin A deficiency (VAD) causally influences oxylipin metabolism and liver health remains unclear. **Methods:** C57BL/6J mouse pups were exposed to either a vitamin A-deficient (VD) or vitamin A-replete (VR) AIN-93G-based diet during the fetal and suckling periods, and they weremaintained on the same diet from weaning (3 weeks of age) to 9 weeks of age. Oxylipin composition in plasma, liver and cerebral tissues was analyzed by liquid chromatography–mass spectrometry. Hepatic and cerebral expressions of genes involved in inflammation, phospholipid and PUFA catabolism, and oxylipin synthesis were analyzed using RT-qPCR. **Results:** Dietary deprivation induced severe VAD, which significantly altered 21 oxylipins in the liver and 34 oxylipins in the cerebrum, but did not affect the plasma oxylipin profile. In the liver, all altered oxylipins were elevated by VAD, the majority being ω-6-derived species with pro-inflammatory properties. In contrast, 27 altered oxylipins were lower in the VD cerebrum, including more ω-3-derived species. Multivariate analysis identified 11,12-EpETrE, 8,9-EpETrE, and 20-HETE as key hepatic oxylipins distinguishing VAD. VAD also altered hepatic expression of genes involved in membrane phospholipid remodeling (*PNPLA8*, *PLA2G6*, *LPCAT3*), and oxylipin metabolism (*ALOX5*, *EPHX2*), and it upregulated inflammatory signaling in the liver only, while fibrosis markers (*TGFB1*, *COL1A1*) remained unchanged. **Conclusions:** These findings demonstrate that early-life VAD is associated with tissue-specific alterations in oxylipin metabolism and hepatic inflammatory responses.

## 1. Introduction

Vitamin A deficiency (VAD) remains one of the most prevalent micronutrient deficiencies in low- and middle-income countries, where it disproportionately affects young children and pregnant women [1]. Beyond its well-studied roles in fetal development, vision and immunity, vitamin A also regulates lipid metabolism, including the synthesis and remodeling of membrane phospholipids and their polyunsaturated fatty acids (PUFAs) [2]. Chronic VAD has been shown to alter tissue PUFA composition and desaturase activity in rodent models [3,4,5,6].

Membrane-bound PUFAs are primary substrates for oxylipins, a large class of oxygenated lipid mediators generated by cyclooxygenases (COXs), lipoxygenases (LOXs), and cytochrome P450 enzymes, as well as non-enzymatic oxidation [7,8]. Oxylipins primarily act through G protein-coupled receptors to regulate inflammation, immune responses, vascular tone, and cell proliferation [9,10,11]. Their production is tightly controlled by PUFA availability and oxygenase activity [8,12,13,14], and may also involve phospholipid remodeling enzymes such as phospholipase A2 (PLA2) and lysophosphatidylcholine acyltransferases (LPCATs) [15].

Although retinoids regulate PUFA metabolism, little is known about their effects on oxylipin synthesis. Limited evidence suggests that retinoic acid (RA) modulates PLA2 activity, alters prostaglandin production, and transcriptionally regulates oxylipin-synthesizing enzymes, such as COXs and LOXs, likely through its interaction with nuclear receptors (e.g., RARs, RXRs and PPARs) [16,17,18,19,20,21]. These observations suggest that VAD may alter oxylipin metabolism at multiple regulatory nodes. Both VAD and abnormal oxylipin profiles have been independently linked to the development of metabolic disorders characterized by chronic inflammation and dysregulated lipid metabolism, such as obesity, diabetes, or non-alcoholic fatty liver disease (NAFLD) and non-alcoholic steatohepatitis (NASH) [6,22,23,24,25]. However, the relationship between VAD, oxylipin dysregulation, and metabolic disease remains unclear. VAD and oxylipin signaling have also been implicated in cognitive impairments in early life [26,27] as well as neuroinflammation and neurodegenerative diseases [28]. Since PUFA profiles differ markedly between the brain and peripheral organs [29,30], it is imperative to delineate tissue-specific oxylipin profiles in response to early-life VAD. In the same early-life VAD mouse model, we previously observed the depletion of plasma and hepatic retinol together with an abnormal gait and impaired Rotarod performance, indicating a dysfunctional motor phenotype following early-life vitamin A depletion [27]. Therefore, the brain was included in the present study as a biologically relevant tissue to determine whether early-life VAD was accompanied by local oxylipin remodeling in a PUFA-enriched organ involved in neurodevelopment and motor regulation. To date, only one clinical study has directly examined vitamin A status in relation to oxylipin metabolism. The study reported lower plasma arachidonic acid (AA)- and eicosapentaenoic acid (EPA)-derived oxylipins in lactating women with a low vitamin A status (plasma retinol <0.8 μmol/L) compared with those with adequate vitamin A (>1.05 μmol/L) [31]. Building on our previous work establishing a robust mouse model of early-life VAD [27], this exploratory study characterized the changes in oxylipin profiles in the plasma, liver, and brain, as well as the accompanying hepatic inflammatory and fibrotic signaling in the context of VAD.

## 2. Materials and Methods

### 2.1. Animal Experiment

The experimental design and animal procedures were reviewed and approved by the Institutional Animal Care and Use Committee of the University of California, Davis (Protocol # 22722, Approval date: 17 March 2022). The details of the experimental design have been previously described [27]. Briefly, C57BL/6J mouse pups (*n* = 8–11/treatment, male-to-female ratio = 1–1.2) were born to dams (*n* = 3–4 dams/treatment) fed either a vitamin A-replete (4000 IU/kg, VR) or deficient (0 IU/kg, VD) diet from breeding until the end of lactation. After weaning at 3 weeks of age, the pups remained on their respective maternal diets until 9 weeks of age. Ingredient and nutrient composition of experimental diets (Dyets, Bethlehem, PA, USA) are provided in Tables S1 and S2. Mouse pups were group-housed after weaning (*n* = 2–4/cage). Liver, brain, and plasma samples were collected at euthanasia. Tissue samples from randomly selected offspring were snap-frozen in liquid nitrogen, and all samples were stored at −80˚C until analysis. Growth and behavioral phenotypes and retinol status have been previously reported [27].

### 2.2. Oxylipin Analysis

Non-esterified oxylipins were extracted and analyzed as previously described [32,33]. Briefly, 50 mg of brain (posterior left cerebral hemisphere) and liver tissue or 100 µL of plasma was mixed with 10 μL of an antioxidant solution (0.02% butylated hydroxytoluene, BHT, 0.02% triphenylphosphine, and 0.1% EDTA), 20 μL of a surrogate solution, and 400 μL of ice-cold methanol containing 0.1% acetic acid and 0.1% BHT. The surrogate solution was a mixture of deuterated internal standards including d4 PGF1α, d4 TXB2, d4 PGE2, d4 LTB4, d11 14,15 DiHETrE, d6 20 HETE, d8 9 HODE, d8 5 HETE, and d11 11,12 EpETrE (Cayman Chemical, Ann Arbor, MI, USA). Tissues were homogenized using an MM301 ball mill (30 Hz, 10 min; Retsch Gmbh, Haan, Germany), and all samples were stored at −80˚C for 24 h. On the following day, supernatants were collected after centrifugation (16,000× *g* at 4˚C for 10 min). Pellets were washed with 100 μL of ice-cold methanol (0.1% acetic acid, 0.1% BHT) and 1.5 mL of distilled water. Solid-phase extraction (SPE) was performed using a Waters Oasis HLB 3cc cartridge (Waters, Milford, MA, USA) preconditioned with ethyl acetate (2 mL), methanol, and 95:5 water:methanol (0.1% acetic acid). Samples were washed and dried under a low vacuum pressure for 20 min. Oxylipins were eluted with 0.5 mL of methanol and 1.5 mL of ethyl acetate into tubes containing 10 μL of 30% glycerol in methanol (trap solution), then dried via SpeedVac concentrator (Neutec Group Inc., Farmingdale, NY, USA) (2 h). Residues were reconstituted in 50 μL of a secondary internal standard solution (200 nM 1-cyclohexyl-dodecanoic acid urea, CUDA), vortexed, and ultra-filtered (0.2 μm, 20 min). Analysis was conducted via liquid chromatography–tandem mass spectrometer (Agilent 1200 SL, Agilent Corporation, Palo Alto, CA, USA; 4000 Qtrap tandem mass spectrometer with an electrospray source (Turbo V), Applied Biosystems, Waltham, MA, USA) using a Pursuit Plus C18 column (2.0 × 150 mm, 5 μm, Varian Inc. Palo Alto, CA, USA). CUDA was used to monitor injection variations and instrument consistency. Mobile phase A was water (0.1% glacial acetic acid); mobile phase B consisted of acetonitrile:methanol (84:16, 0.1% glacial acetic acid). Gradient elution was performed at 400 μL/min. Quantification of oxylipins was performed by normalizing the peak area of each oxylipin to the peak area of the class-specific deuterated internal standard and the wet tissue weight. *n* = 10 mice per treatment were used for the oxylipin analysis.

### 2.3. Real-Time Quantitative PCR (RT-qPCR)

Total RNA extraction and RT-qPCR were performed as previously described [34]. Briefly, total RNA was extracted from the right cerebral hemisphere and liver using TRIzol (Invitrogen, Waltham, MA, USA) and quantified via a NanoDrop (Thermo Fisher Scientific, Waltham, MA, USA). cDNA was synthesized using the High-Capacity cDNA Reverse Transcription Kit (Applied Biosystems, Waltham, MA, USA). Target genes include enzymes for PUFA mobilization (*PLA2G4A*, *PLA2G6*, and *PNPLA8*), esterification (*LPCAT3*) and oxylipin synthesis (*ALOX5*, *ALOX8*, *CYP2J9* and *EPHX2*), a retinol transporter (*STRA6*), inflammation markers (*TNF*, *IL6* and *CRP*), the antioxidant marker (*GPX4*), and fibrosis markers (*COL1A1* and *TGBF1*). The RT-qPCR reactions were performed in duplicate using the PowerUp SYBR Green Master Mix in 20 µL reactions on a QuantStudio 3 system (Applied Biosystems). Relative expression was calculated using the comparative Ct method and normalized to *GAPDH*. Primer sequences are listed in Table S3. *n* = 8–11/mice per treatment were used for the RT-qPCR analysis.

### 2.4. Histological Assessment and ELISA Analysis

Liver tissues (*n* = 5 for VD, *n* = 3 for VR) were fixed in 4% paraformaldehyde, paraffin-embedded, and sectioned at 5 µm. Sections were stained with Masson’s trichrome for qualitative assessment of histology and fibrosis. Briefly, slides were incubated in Bouin’s solution (55˚C, 1 h), followed by Weigert’s Hematoxylin, Biebrich Scarlet–Acid Fuchsin (5 min), a phosphomolybdic–phosphotungistic acid solution (30 s), and an Aniline Blue solution (10 min), with intermediary rinses. Following a 1% acetic acid incubation, the slides were dehydrated and coverslipped. The histological assessment of fibrosis was evaluated qualitatively based on the severity of periportal fibrosis and the presence of fibrous septa.

### 2.5. ELISA Analysis

Liver tissues (~100 mg) were homogenized in ice-cold RIPA buffer containing protease inhibitor cocktail (Sigma-Aldrich, St. Louis, MO, USA). Total protein was extracted and analyzed for concentration using the Bradford protein assay (Thermo Fisher Scientific). The diluted protein samples were analyzed for TNFα and TGFβ1 concentrations using mouse DuoSet ELISA kits (R&D Systems, Minneapolis, MN, USA). The concentration of each cytokine was normalized against the total protein concentration.

### 2.6. Statistical Analysis

Data analysis was conducted using Prism (version 10, GraphPad, San Diego, CA, USA) and MetaboAnalyst (v.6.0, https://www.metaboanalyst.ca/) for plasma and liver retinol concentrations, gene expression and oxylipin data. Because most plasma and liver retinol concentrations in the VD mice were below the limit of detection, the retinol data were analyzed using the nonparametric Mann–Whitney U test and are reported as the median and interquartile range (IQR). Relative gene expression data were verified for homoscedasticity and normal distribution, and they were analyzed using the unpaired *t*-test for the treatment effect. Statistical significance was set at *p* < 0.05, with trends toward significance defined as *p* < 0.1. Internal standard- and tissue weight-normalized oxylipin mass spectrometry data were uploaded to MetaboAnalyst (v.6.0) and further normalized through log10 transformation to comply with a normal distribution. The normalized data were then subjected to univariate (unpaired *t*-test) and multivariate analyses. For the univariate analysis, a significant treatment effect was declared when an oxylipin concentration changed > 2 fold (|Log_2_(FC)| > 1) and had an FDR-adjusted *p*-value < 0.05 (−Log_10_(*p*-value) > 1.3). Multivariate analyses included principal component analysis (PCA), partial least squares discriminant analysis (PLS-DA), and heatmap clustering analysis. The statistical significance of the group clustering in the PCA plot was evaluated using PERMANOVA and sample distributions were computed using the Euclidean distance. Variable importance in projection (VIP) scores were calculated for the PLS-DA. The number of components for classification was selected based on the highest predictive ability determined by 5-fold cross-validation. All results were presented as means and standard errors.

## 3. Results

### 3.1. Plasma and Hepatic Retinol Concentration

Plasma and hepatic retinol concentrations were previously reported by Arballo et al. (2024) [27] and were reanalyzed here using a nonparametric test without accounting for sex, because sex was not significant in the initial model (Table 1). As shown in Table 1, the plasma retinol levels were significantly lower in the VD group compared to the VR group (*p* < 0.001). Hepatic retinol was undetectable in all the VD mice (limit of quantification between 0.4 and 1.0 pmol/L [35]), while the VR group had a mean concentration of 0.274 µmol/g tissue (*p* < 0.001).

### 3.2. Hepatic Oxylipin Profile

**Figure 1:**
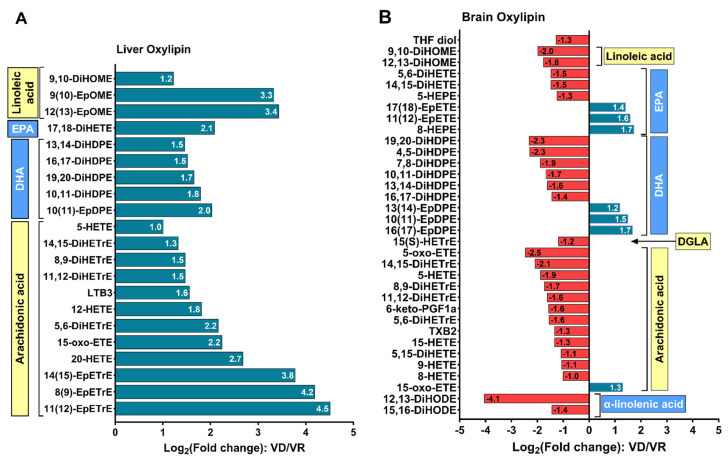
Log_2_(fold change) in oxylipins significantly affected (FDR-adjusted *p*-value < 0.05) by early-life vitamin A deficiency in liver (**A**) and cerebrum (**B**). The polyunsaturated fatty acid (PUFA) precursors of oxylipins were annotated with ω-6 and ω-3 PUFAs highlighted in yellow and blue, respectively. DHA, docosahexaenoic acid; EPA, eicosapentaenoic acid; DGLA, Dihomo-γ-linolenic acid. VR, vitamin A replete (*n* = 10); VD, vitamin A deficiency (*n* = 10).

LC-MS analysis detected 39 oxylipins in the liver, of which 21 were significantly affected by the treatment group, with all showing higher levels in the VD group compared to the VR group (Figure 1A and Figure S1A and Table S4). Among these, 12 oxylipins were derived from arachidonic acid (AA), while docosahexaenoic acid (DHA), linoleic acid (LA), and eicosapentaenoic acid (EPA) contributed to five, three, and one altered oxylipin, respectively (Figure 1). The VD treatment induced a coordinated increase in oxylipins produced via both the lipoxygenase and cytochrome P450 (CYP450) pathways.

**Figure 2:**
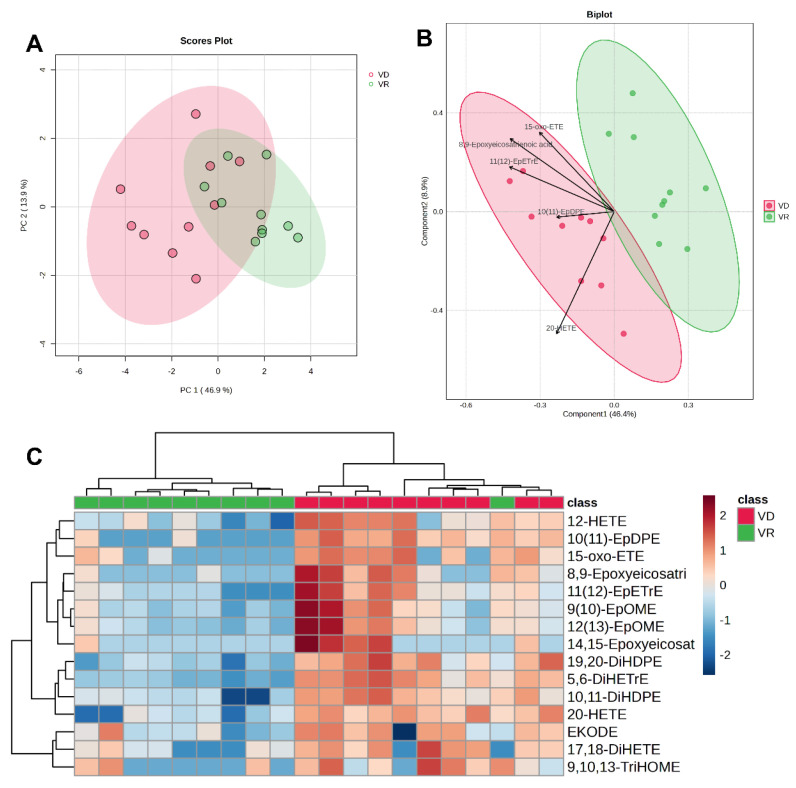
Distinct hepatic oxylipin profiles associated with early-life vitamin A deficiency. (**A**) Principal component analysis (PCA) and (**B**) partial least squares discriminant analysis (PLS-DA) biplots demonstrated group-specific separation along the top two components. The PLS-DA biplot highlighted the top 5 oxylipins contributing to group separation. (**C**) Heatmap showing hierarchical clustering of samples based on the variable importance in projection (VIP) scores of the top 15 oxylipins. VR, vitamin A replete (*n* = 10); VD, vitamin A deficiency (*n* = 10).

Principal component analysis (PCA) revealed a partial but statistically significant separation of samples by treatment along PC1, which accounted for 47% of the total variance (*p* = 0.001; Figure 2A). This separation was further confirmed and enhanced by supervised partial least squares discriminant analysis (PLS-DA), which highlighted the top five oxylipins contributing to group classification based on their regression coefficients, as shown in the biplot (Figure 2B). The predictive performance of the PLS-DA model was evaluated by cross-validation, and the corresponding R^2^ and Q^2^ values across different latent components are presented in Figures S1B and S2B for hepatic and plasma oxylipins, respectively. These five features corresponded to those with the highest variable importance in projection (VIP) scores (VIP ≥ 1.4; Figure S1C), identifying 11(12)-EpETrE, 8(9)-EpETrE (8,9-epoxyeicosatrienoic acid), and 20-HETE as the most distinguishing metabolites associated with the VD group. The Q^2^ values remained consistently above 0.5 across different model components, indicating the strong predictive reliability of the PLS-DA model (Figure S1B). Hierarchical clustering analysis based on the 15 oxylipins with the highest VIP scores showed clear clustering of samples by treatment, except for one VR sample that grouped with the VD cluster (Figure 2C).

### 3.3. Brain Oxylipin Profile

In the cerebrum, LC-MS detected 77 oxylipins, of which 34 were significantly affected by the VD treatment, as determined by unpaired *t*-tests (Figure 1B and Figure S2A, and Table S5). In contrast to the liver, where all altered oxylipins were elevated in the VD groups, only seven of the 34 altered cerebral oxylipins were higher in the VD group, while the remaining 27 were significantly lower compared to the VR group (Figure 1B). The affected oxylipins were derived from AA (13), DHA (9), EPA (6), ALA (2), LA (2), and DGLA (1). Most of these were downstream products of the lipoxygenase and CYP450 pathways, with two involved in thromboxane and prostacyclin biosynthesis.

**Figure 3:**
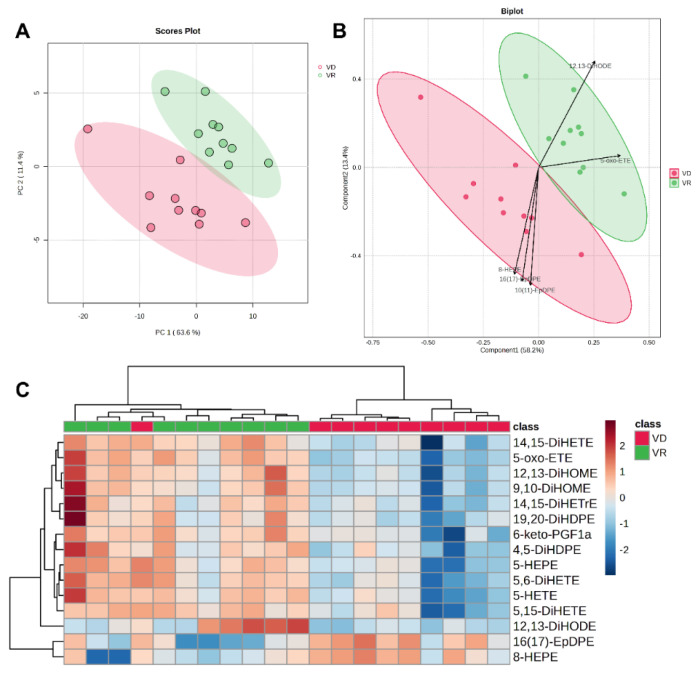
Distinct cerebral oxylipin profiles associated with early-life vitamin A deficiency. (**A**) PCA and (**B**) PLS-DA biplot illustrated group-specific separation along the top two components. The PLS-DA biplots highlighted the top five oxylipins contributing to group separation. (**C**) Heatmap showing hierarchical clustering of samples based on the VIP scores of the top 15 oxylipins. VR, vitamin A replete (*n* = 10); VD, vitamin A deficiency (*n* = 10).

The PCA of the cerebral oxylipin profile revealed a distinct clustering of samples by treatment, with minimal overlap between the 95% confidence ellipses (Figure 3A). Principal components 1 and 2 explained 63.6% and 11.4% of the total variance, respectively. Similarly, the supervised PLS-DA confirmed the group separation, with components 1 and 2 together accounting for 71.6% of the variance (Figure 3B). Cross-validation indicated an optimal predictive performance using two components (Figure S2B). The PLS-DA biplot highlighted the top five contributing oxylipins based on regression coefficients, with 12,13-DiHODE, 5-oxo-ETE, and 8-HEPE also having VIP scores greater than 1.5 (Figure S2C). Hierarchical clustering analysis based on the 15 oxylipins with the highest VIP scores further supported the grouping by treatment, with one VD sample clustering with the VR grouping (Figure 3C).

### 3.4. Plasma Oxylipin Profile

In contrast to its significant impact on the hepatic and cerebral oxylipin profiles, the VD diet did not alter any of the 38 oxylipins detected in the plasma (Table S6).

### 3.5. Hepatic Gene and Protein Expression and Histopathology

**Figure 4:**
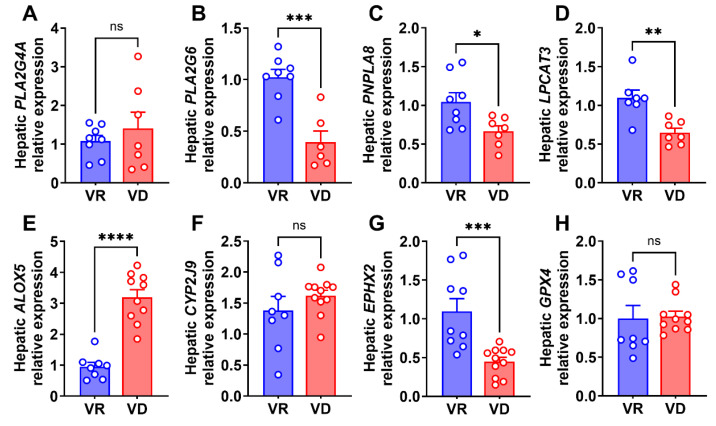
Effect of early-life vitamin A deficiency on relative gene expression in liver. Panels A–H show the relative mRNA expression levels of (**A**) cytosolic phospholipase A_2_ (*PLA2G4A*), (**B**) calcium-independent phospholipase A_2_ (*PLA2G6*), (**C**) palatin-like phospholipase domain-containing protein 8 (*PNPLA8*), (**D**) lysophosphatidylcholine acyltransferase 3 (*LPCAT3*), (**E**) arachidonate lipooxygenase-5 (*ALOX5*), (**F**) cytochrome P450, family 2, subfamily J, polypeptide 9 (*CYP2J9*), (**G**) soluble epoxide hydrolase 2 (*EPHX2*), and (**H**) glutathione peroxidase 4 (*GPX4*) VR, vitamin A replete (*n* = 8–9); VD, vitamin A deficiency (*n* = 10–11). **** *p* < 0.0001; *** *p* < 0.001; ** *p* < 0.01; * *p* < 0.05; ns, not significant (*p* > 0.05).

*PLA2G4A*, *PLA2G6*, *PNPLA8*, *LPCAT3*, *ALOX5*, *ALOX8*, *PTGS2* (*COX2*), *CYP2J9*, and *EPHX2* encode key enzymes regulating oxylipin metabolism in tissues. *PTGS2* (*COX2*) and *ALOX8* were either undetectable at a cycle threshold (Ct) 35 or detected with high Ct values (>33 cycles) in most samples in the RT-qPCR reactions. While *PLA2G4A* and *CYP2J9* expression remained unaffected by the treatment (Figure 4A,F,H), the VD mice had significantly lower hepatic expression of *PLA2G6*, *PNPLA8*, *LPCAT3*, as well as *EPHX2* and higher expression of *ALOX5* compared to the VR group (*p* < 0.05; Figure 4B–E,G). Although glutathione peroxidase 4 (*GPX4*) plays a critical role in tissue antioxidative defense, its mRNA expression was not affected by the VD diet (Figure 4H).

**Figure 5:**
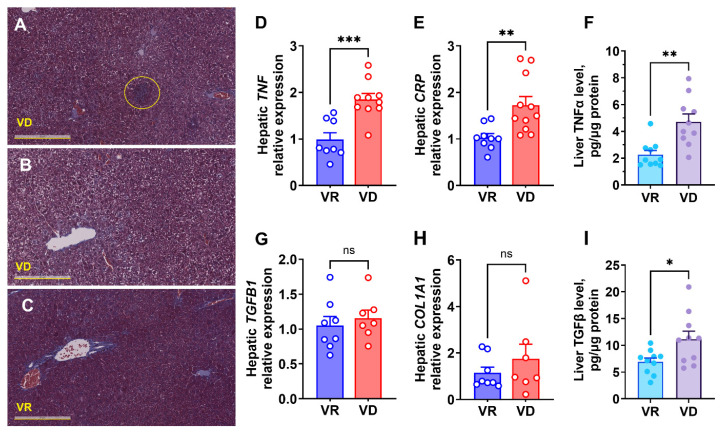
Effects of early-life vitamin A deficiency on liver histology and hepatic expression of inflammation and fibrosis marker genes. (**A**–**C**): Representative sample images of Masson’s trichrome-stained liver sections from VD (**A**,**B**) and VR (**C**) mice. The scale bar in images denotes 200 µm. (**D**–**I**): Relative mRNA expression of tumor necrosis factor (*TNF*, (**D**)), C-reactive protein (CRP, (**E**)), transforming growth factor beta-1 (*TGFB1*, (**G**)) and collagen type I, alpha 1 (*COL1A1*, (**H**)), and liver TNFα (**F**) and TGFβ1 (**I**) concentrations. VR, vitamin A replete (*n* = 3 for histology and 8 for gene expression); VD, vitamin A deficiency (*n* = 5 for histology and 8 for gene expression). ns, not significant (*p* > 0.05). *** *p* < 0.001; ** *p* < 0.01; * *p* < 0.05; ns, not significant (*p* > 0.05).

Only minimal collagen deposition was observed, with no histological signs consistent with advanced fibrosis or cirrhosis. (See representative sample images in Figure 5A–C). The VAD diet upregulated *TNF* and *CRP* expression, and it increased TNFα and TGFβ1 concentrations in the liver tissues (*p* < 0.05) but it did not affect the gene expression of fibrotic markers (*COL1A1* and *TGFB1*; Figure 5D–I).

### 3.6. Cerebral Gene Expression

**Figure 6:**
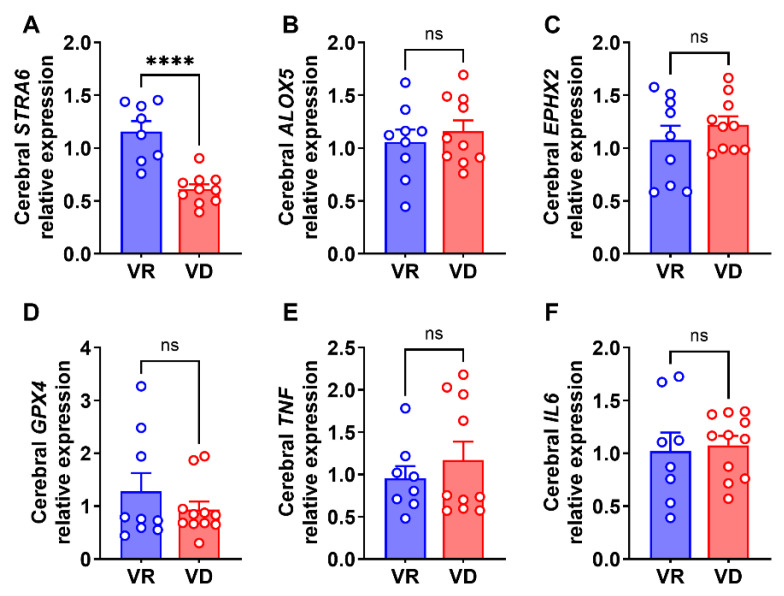
Effect of early-life vitamin A deficiency on relative gene expression in cerebrum. Panels (**A**–**F**) show the relative mRNA expression levels of (**A**) stimulated by retinoic acid 6 (*STRA6*), (**B**) arachidonate 5-lipoxygenase (*ALOX5*), (**C**) soluble epoxide hydrolase 2 (*EPHX2*), (**D**) glutathione peroxidase 4 (*GPX4*), (**E**) tumor necrosis factor alpha (*TNF*), and (**F**) interleukin 6 (*IL6*), respectively. VR, vitamin A replete (*n* = 8–9); VD, vitamin A deficiency (*n* = 10–11). **** *p* < 0.0001; ns, not significant (*p* > 0.05).

*STRA6* encodes a cell membrane receptor for retinol-binding protein and mediates retinol uptake in the brain and other organs [36,37]. The mRNA expression of *STRA6* was used as a surrogate marker for brain retinol status. The VD diet resulted in the downregulation of *STRA6* in the cerebrum (*p* < 0.0001; Figure 6A). Unlike in the liver, neither *ALOX5* nor *EPHX2* was affected by the VD diet in the cerebrum (Figure 6B,C). Similarly, neither *GPX4* nor the genes encoding pro-inflammatory cytokines (*TNF* and *IL6*) were influenced by the VD diet (Figure 6D–F).

## 4. Discussion

### 4.1. VAD Modulates Hepatic Oxylipin Metabolism Toward a Pro-Inflammatory Profile

VAD remains a global health challenge, particularly in low- and middle-income countries [38]. Diminished vitamin A stores are observed in hepatic metabolic disorders such as NAFLD, but whether this is a contributing factor or a consequence of the disease remains unclear [23,39,40]. While prior studies demonstrated that VAD disrupts phospholipid and PUFA composition [5,41,42,43,44], our model showed, for the first time, that the depletion of hepatic vitamin A significantly affected oxylipin profiles. Strikingly, all 21 hepatic oxylipins altered by VAD were elevated, with most derived from ω-6 PUFAs (LA and AA), particularly AA-derived eicosanoids (Figure 1A). Many of these oxylipins, such as 5-HETE, 12-HETE, and 20-HETE, are well-characterized mediators of inflammation, oxidative stress, and vascular dysfunction [45,46,47,48,49]. Likewise, LA-derived EpOMEs and their DiHOME metabolites catalyzed by cytochrome P450 and soluble epoxide hydrolase (sEH), respectively, were also elevated in the VD mice. Both 9,10-EpOME and 9,10-DiHOME were shown to activate NF-κB and AP-1 signaling and induce oxidative stress and inflammation [50,51]. In contrast, some ω-3 PUFA-derived oxylipins, such as DHA-derived EpDPE and its diol metabolites (DiHDPEs), exhibit anti-inflammatory and vasoprotective properties by inhibiting platelet aggregation and thromboxane synthesis [7,8]. Together, these findings support the notion that VAD shifts hepatic oxylipin metabolism toward a more pro-inflammatory profile.

Within the current study, the hepatic accumulation of AA- and LA-derived oxylipins, together with inflammatory gene and protein changes, suggests that hepatic vitamin A deficiency may shift hepatic lipid mediator metabolism toward a state that is permissive for inflammatory activation. Several mechanistic routes may therefore converge to explain the hepatic oxylipin phenotype. First, as aforementioned, VAD may alter the pool of available PUFA substrates by changing membrane fatty acid composition, desaturase activity, and phospholipid remodeling [3,4,15,52,53,54,55,56]. Because oxylipin synthesis depends on both the liberation of PUFAs from membrane phospholipids and their reincorporation back into membranes, the disruption of either process could alter the amount of free substrate available for oxygenase pathways [57,58]. The release of PUFAs from membrane phospholipids is another critical step in oxylipin synthesis. Retinoid supplementation in human synovial fluid inhibits the activity of PLA2, an enzyme that preferentially cleaves AA from membrane phospholipids, which may modulate AA-derived oxylipin synthesis [17,59]. In the present study, however, VAD reduced the hepatic gene expression of calcium-independent PLA2 enzymes (*PNPLA8* and *PLA2G6*) despite increasing AA-derived oxylipins. Therefore, the increased hepatic AA-derived oxylipins are unlikely to be explained solely by increased PLA2 gene expression and may instead reflect additional mechanisms affecting substrate availability.

One plausible mechanism is the reduced AA reincorporation into phospholipids through LPCAT3. Hepatic *LPCAT3* expression was decreased in the VD mice in this study, which may reduce the reincorporation of AA into membrane phospholipids and thereby increase the pool of free AA available for COX, LOX, CYP450, and sEH pathways. This interpretation is consistent with prior evidence: VAD was shown to increase the AA concentration in liver tissue homogenates in a rodent model [43,44]; furthermore, all-trans retinoic acid (ATRA) upregulates LPCAT3 mRNA and enzyme activity in fibroblasts [56], and *LPCAT3* knock-out reduces arachidonate incorporation into the cell membrane in mice [15]. Thus, the prior literature and the present findings support a plausible mechanism in which VAD increases AA-derived oxylipin production not through greater PUFA liberation by PLA2, but through impaired LPCAT3-dependent reacetylation and membrane retention of AA.

Changes in oxylipin-producing and oxylipin-metabolizing enzymes may also contribute to the hepatic oxylipin profile observed. Retinoid signaling provides a plausible upstream mechanism for these enzyme-level changes, as retinoic acid has been shown to modulate *COX* expression and prostaglandin production in vitro and in vivo, regulate CYP450 isoforms involved in fatty acid oxidation, and interact with nuclear receptor pathways that control lipid-metabolizing oxygenases [16,18,20,60,61]. In the present study, the increase in hepatic *ALOX5* together with elevated 5-HETE is consistent with this framework, suggesting that VAD may not only alter precursor PUFA availability but also reshape the enzymatic routing of those substrates toward specific oxylipin products. However, the discordance between reduced *EPHX2* expression and elevated downstream diols indicates that gene expression alone does not fully explain the oxylipin phenotype observed [62,63], and that oxygenase activity, substrate flux, and cell-specific regulation should be examined in future studies.

Collectively, these findings show that VAD induces a hepatic oxylipin profile enriched in AA- and LA-derived pro-inflammatory lipid mediators. Additional studies measuring precursor PUFA pools, phospholipid composition, oxygenase activity, and oxylipin flux will be required to define the causal mechanisms. Thus, our data support the interpretation that VAD shifts hepatic oxylipin metabolism toward a pro-inflammatory biochemical state, and we highlight several potential mechanisms here.

### 4.2. VAD Induces Hepatic Inflammation Without Fibrosis

Consistent with the pro-inflammatory profile of hepatic oxylipins observed in this study, VAD upregulated TNFα (mRNA and protein levels) and *CRP* gene expression, corroborating elevated hepatic inflammation and stress. However, this elevated inflammation was not paralleled with a consistent induction of canonical fibrotic marker genes (*TGFB1*, *COL1A1*) or clear histological signs of fibrosis, despite an increase in TGF-β protein in the VD livers. It should be noted that the histological signs of fibrosis were assessed qualitatively, due to the limited number of samples available for Masson’s trichrome staining (VR, *n* = 3; VD, *n* = 5). The present model appears to capture an earlier inflammatory and lipid mediator remodeling state, in which VAD is sufficient to elevate hepatic pro-inflammatory oxylipins and inflammatory markers, but not sufficient, within the experimental window, to induce overt collagen deposition or fibrotic gene expression. This distinction strengthens the developmental programming interpretation: VAD may create a sensitized hepatic environment that precedes fibrosis and may require a second metabolic or inflammatory challenge to progress toward chronic liver pathology, such as an obesogenic environment. For example, diminished hepatic vitamin A stores have been noted in the progression of steatosis and fibrosis in both clinical and experimental settings [23,39,40]. For instance, in a cohort study of 68 morbidly obese individuals, hepatic retinol levels were inversely correlated with the severity of steatosis [64]. Similarly, obese mouse models induced by either dietary or genetic manipulations exhibit reduced hepatic vitamin A stores [65]. A hallmark of hepatic fibrosis is the activation of hepatic stellate cells (HSCs), characterized by a transition from a quiescent, vitamin A-storing state to an active, collagen-producing state, thereby promoting fibrosis [39,66]. This distinction is important because vitamin A metabolism is deeply embedded within the same hepatic cell populations that participate in inflammatory and fibrogenic liver disease. Therefore, a diminished hepatic vitamin A status is not merely a marker of altered nutrient storage, but it is closely tied to the cellular architecture of liver injury, inflammation, and fibrogenesis.

This connection is particularly relevant because vitamin A metabolism, inflammatory signaling, oxylipin production, and fibrogenic activation are distributed across interacting hepatic cell populations. Kupffer cells express vitamin A-related metabolic function and produce inflammatory oxylipins, including prostaglandin E2, following immune stimulation, while retinoids can modulate Kupffer cell cytokine release [67,68,69,70,71,72,73]. Hepatocytes regulate systemic retinol transport through retinol-binding protein production and participate in prostaglandin metabolism, including conversion of PGE2 to 15-keto-PGE2 through 15-hydroxyprostaglandin dehydrogenase [74,75,76,77]. HSCs are central to hepatic vitamin A storage and can induce inflammatory oxylipin signaling during activation, including increased 5-lipoxygenase signaling in activated compared with quiescent HSCs, paralleling the increased hepatic Alox5 gene expression and 5-LOX-derived cognate oxylipins observed in the VD mice in our study [78,79,80,81]. Emerging evidence further supports a role for oxylipins in liver metabolic disorders. Plasma oxylipins (e.g., 11,12-DiHETrE, 12-HETE, and 14,15-DiHETrE) were positively associated with fibrosis in NASH patients [82]. Conversely, inhibiting *ALOX-5* expression alleviated steatosis in high-fat-fed rats [83], extending this pathway from inflammatory oxylipin signaling to a causal role in metabolic liver injury. These findings are consistent with, and provide strong support for, a literature-based framework in which VAD promotes early inflammatory oxylipin remodeling within a hepatic retinoid–oxylipin network. However, because this study did not directly manipulate individual oxygenase or LPCAT3 pathways or test progression under a secondary metabolic challenge, these data should be interpreted as mechanistic support rather than definitive causal proof. Future studies will be needed to determine whether VAD-induced oxylipin remodeling sensitizes the liver to subsequent fibrogenic or metabolic injury. Although this phenotype did not progress to overt fibrosis within the present experimental window, the concurrent elevation of pro-inflammatory oxylipins, TNFα (mRNA and protein), *CRP*, TGFβ protein, and *ALOX5* gene expression supports the interpretation that VAD establishes an inflammatory hepatic state that may become pathologically consequential when combined with additional metabolic or inflammatory stressors, such as a high-fat intake [84].

### 4.3. VAD Produces Tissue-Specific Oxylipin Responses in Brain and Plasma

Direct quantification of cerebral retinoids is technically challenging because of the high lipid content in the brain, particularly complex lipids (e.g., phospholipids and sphingolipids). Instead, we assessed *STRA6* expression, which was reduced in the VD mice, indicating lower cerebral retinol levels [36,37]. Unlike in the liver, most cerebral oxylipins altered by VAD were decreased, especially ω-3-derived metabolites. VAD did not induce neuroinflammation or alter mRNA expression of oxylipin-synthesizing enzymes. These findings suggested tissue-specific responses in oxylipin metabolism, which may be due, in part, to differences in PUFA composition between the brain and liver. As shown in our mouse model, the brain contains lower levels of AA and LA compared to the liver [29,85]; furthermore, the developing brain incorporates DHA and AA at much higher rates than other organs to support neurodevelopment [30,86]. The functional implications of the observed changes in cerebral oxylipins are unclear. Some oxylipins with anti-inflammatory and neuroproliferative properties (e.g., DHA-derived EpDPE) were increased [87,88], while others, such as EPA-derived 5-HEPE, which also exhibits neuroprotection against cytokine-induced apoptosis, were decreased by VAD [89]. The mixed changes indicate that VAD causes a nuanced, rather than a uniform, shift in cerebral oxylipins. Moreover, oxylipin-synthesizing enzymes display cell- and tissue-specific expression patterns [90], which may also contribute to the varied responses in the brain. Thus, the cerebral analyses were motivated by the reproducible motor phenotype previously observed in this early-life VAD model, but the present oxylipin and gene expression data do not support a simple neuroinflammatory interpretation. Instead, they suggest that VAD produces a localized cerebral oxylipin remodeling that is directionally distinct from the hepatic pro-inflammatory response.

In contrast, the plasma oxylipin profiles were unaffected despite severe VAD. This differs from the findings in a pilot study of Filipino women, wherein low vitamin A status was associated with lower plasma concentrations of AA- and EPA-derived oxylipins, as well as lysophospholipids and sphingolipids [31], highlighting a systemic impact of marginal VAD on oxylipin and phospholipid metabolism in humans. This discrepancy may reflect differences in species and physiological stages. The absence of significant plasma changes despite marked hepatic and cerebral alterations may suggest compartmentalized oxylipin production, wherein the observed tissue profiles reflect localized rather than systemic changes. However, because the present study did not directly assess oxylipin flux or tissue-to-plasma exchange, this interpretation remains speculative. Findings from the current study underscore that circulating oxylipins may not reliably reflect local tissue metabolism, which is highly compartmentalized [91].

### 4.4. Limitations of the Study

Several limitations should be considered when interpreting these findings. First, the tissue PUFA composition was not measured, preventing the determination of whether the altered oxylipin profiles were driven by changes in precursor fatty acid availability. Second, the oxylipin-producing enzyme activity and protein abundance were not assessed; therefore, the mechanistic links between gene expression and oxylipin production remain speculative. Third, sex-specific analyses were not performed because the study was not powered for such comparisons. Fourth, the litter effects were not incorporated into the statistical model because this work represents a secondary exploratory analysis of archived samples. Finally, the lack of a vitamin A repletion treatment in the current study prevents the examination of the causal links with the observed changes in the oxylipin profiles and the hepatic inflammatory response. Nevertheless, the study is strengthened by the confirmed vitamin A deficiency, the targeted oxylipin profiling across the brain, liver and plasma and consistent protein and gene expression alterations in inflammation, supporting the tissue-specific effects of early-life VAD in a mouse model.

## 5. Conclusions

Early life VAD resulted in distinct, tissue-specific changes in oxylipin metabolism. In the liver, VAD promoted a pro-inflammatory oxylipin profile and induced inflammation, but it did not cause fibrosis under non-obesogenic conditions. In the brain, VAD produced mixed oxylipin changes without causing neuroinflammation. The plasma oxylipins were unaffected despite significant tissue perturbations. Together, these findings reveal that retinoid–oxylipin interactions are complex and extend beyond gene expression. Future studies should explore whether VAD and the altered oxylipin profiles exacerbate the progression of metabolic diseases when combined with obesogenic stress or chronic inflammation.

## Figures and Tables

**Table 1:** Plasma and hepatic retinol concentrations ^1^.

Analysis	Median (IQR ^2^)		
	VR	VD	*p*-Value
Plasma retinol ^3^, µmol/L	0.575 (0.516–0.704)	N.D. ^5^	<0.001
Liver retinol ^4^, µmol/g wet tissue	0.272 (0.107–0.397)	N.D. ^5^	<0.001

^1^ Plasma and hepatic retinol data were published in (Arballo et al., 2024, [27]) and are reanalyzed without sex in the model using unpaired *t*-test. ^2^ Interquartile range reports the 25th and 75th percentile. ^3^ Plasma samples: VR, *n* = 18; VD, *n* = 12. ^4^ Liver samples: VR, *n* = 8; VD, *n* = 9. ^5^ Not detectable. For plasma and liver retinol concentrations below the limit of detection, a value of 0 was assigned for nonparametric analysis using the Mann–Whitney U test. Consequently, the median and IQR of the VD group were 0 (0–0).

## Data Availability

The original contributions presented in this study are included in the article/Supplementary Materials. Further inquiries can be directed to the corresponding author.

## Supplementary Materials

The following supporting information can be downloaded at: https://www.mdpi.com/article/10.3390/nu18121988/s1, Figure S1: Effect of early-life VAD on hepatic oxylipin profiles; Figure S2: Effect of early-life VAD on cerebral oxylipin profiles; Table S1: Ingredient composition of experimental diets; Table S2: Composition of mineral-vitamin premix; Table S3: The primer sequences; Table S4: Relative change (Log2 fold change) and FDR-adjusted *p*-values of all oxylipins detected in liver; Table S5: Relative change (Log2 fold change) and FDR-adjusted *p*-values of all oxylipins detected in cerebrum; Table S6: Relative change (Log2 fold change) and FDR-adjusted *p*-values of all oxylipins detected in plasma.

## Abbreviations

The following abbreviations are used in this manuscript:

AA	Arachidonic acid
ALA	Alpha-linolenic acid
ALOX5	Arachidonate 5-lipoxygenase
ALOX8	Arachidonate 8-lipoxygenase
COL1A1	Collagen, type I, alpha 1
COX	Cyclooxygenase
CRP	C-Reactive protein
CYP2J9	Cytochrome P450 family 2 subfamily J member 9
DGLA	Dihomo-γ-linolenic acid
DHA	Docosahexaenoic acid
DiHEtrES	Dihydroxy epoxytrienoic acids
DiHETE	Dihydroxyeicosatrienoic acid
DiHOMES	Dihydroxy octadecenoic acids
EET	Epoxyeicosatrienoic acid
EPA	Eicosapentaenoic acid
EPHX2	Epoxide hydrolase 2
ETE	Eicosatrienoic acid
GAPDH	Glyceraldehyde-3-phosphate dehydrogenase
GPHX4	Glutathione peroxidase 4
HETE	Hydroxyeicosatetraenoic acid
IL6	Interleukin 6
LA	Linoleic acid
LOX	Lipoxygenase
PCA	Principal component analysis
PLA2G4A	Phospholipase A2 group IVA
PLS-DA	Partial least squares discriminant analysis
PUFA	Polyunsaturated fatty acids
RA	Retinoic acid
SeH/EPHX2	Soluble epoxide hydrolase
STRA6	Stimulated by retinoic acid 6
TGFB1	Transforming growth factor, beta 1
TNF/TNF-α	Tumor necrosis factor, alpha
VD	Vitamin A deficiency
VR	vitamin A replete
