# Supplementary material for: Early-Life Vitamin A Deficiency Induces Tissue-Specific Oxylipin Remodeling and Hepatic Inflammation
Source: Nutrients. 2026 Jun 19;18(12):1988. doi: 10.3390/nu18121988 (PMC13305949; doi:10.3390/nu18121988)
Supplement: Supplementary file 1 [file nutrients-18-01988-s001.zip › nutrients-4332343-supplementary.pdf]

1 Supplementary Figures

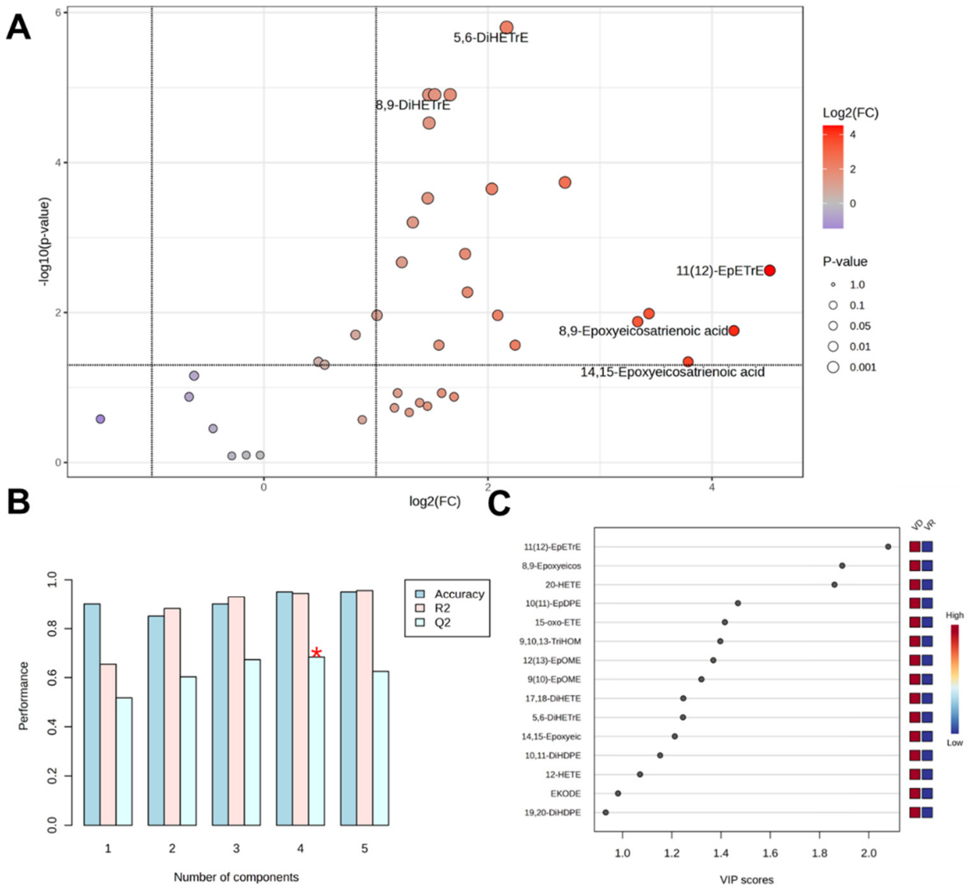

Figure S1. Effect of early-life VAD on hepatic oxylipin profiles. (A) volcano plot showing log2(fold change) and -log10(p-value) of oxylipins detected in liver, (B) cross-validation showing model predictability at different number of components in partial least squares discriminant analysis (PLSDA), (C) Variable importance in projection (VIP) scores of the top 15 hepatic oxylipins. The asterisk indicates the optimal component number selected based on the highest predictive ability from 5-fold cross-validation. VR, vitamin A replete (n = 10); VD, vitamin A deficiency (n = 10)

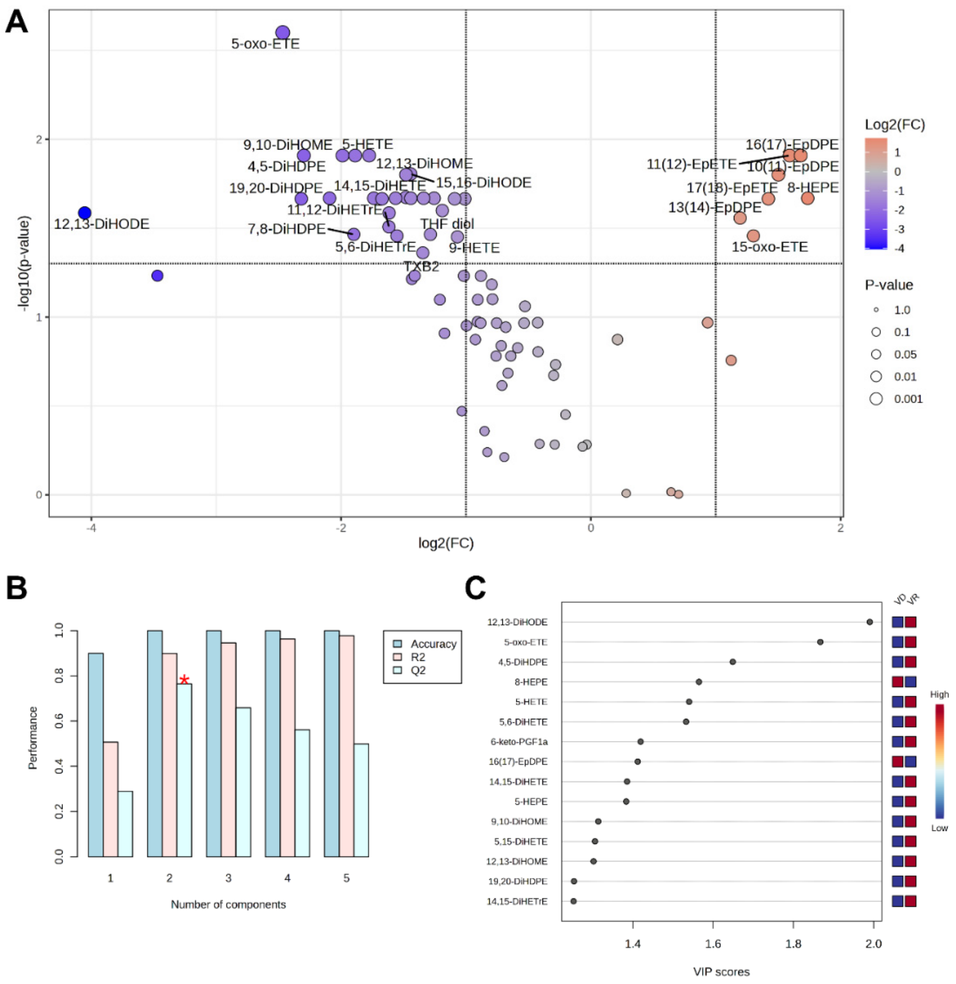

Figure S2. Effect of early-life VAD on cerebral oxylipin profiles. (A) volcano plot showing log2(fold change) and -log10(p-value) of oxylipins detected in cerebrum, (B) cross-validation showing model predictability at different number of components in partial least squares discriminant analysis (PLSDA), (C) Variable importance in projection (VIP) scores of the top 15 cerebral oxylipins. The asterisk indicates the optimal component number selected based on the highest predictive ability from 5-fold cross-validation. VR, vitamin A replete (n = 10); VD, vitamin A deficiency (n = 10)

2 Supplementary Tables

Table S1: Ingredient composition of experimental diets

	Vitamin A-adequate diet (DYET#119343)[a]	Vitamin A-free diet (DYET#119342)[a]
Ingredients	g/kg	g/kg
Vitamin free casein	200	200
L-cystine	3	3
Sucrose	94	98
Cornstarch	397.5	397.5
Dyetrose	132	132
Cottonseed oil with tBHQ	70	70
Microcrystallline cellulose	50	50
Mineral-vitamin premix (no vitamin A)[b]	45.04	45.04
Vitamin A palmitate (1,000 IU/g)	4	0
Choline bitartrate	2.5	2.5
Total	1000	1000

[a.] Diets were manufactured by Dyets Inc (Bethlehem, PA). Ingredient composition has been previously published (DOI: 10.3390/ijms251910806)
[b.] See Table S2 for composition.

Table S2: Composition of mineral-vitamin premix[a]

Mineral compound	Amount in diet, mg/kg	Vitamin compound	Amount in diet, mg/kg
Calcium carbonate	12,495	Niacin	30.0
Potassium phosphate (monobasic)	6,860	Calcium pantothenate	16.0
Potassium citrate·H_2O	2,477.3	Pyridoxine HCl	7.0
Sodium chloride	2,590	Thiamine HCl	6.0
Potassium sulfate	1,631	Riboflavin	6.0
Magnesium oxide	840	Folic acid	2.0
Ferric citrate	43.9	Biotin	0.2
Zinc carbonate	57.75	Vitamin E acetate (500 IU/g)	150.0
Manganese carbonate	22.05	Vitamin B12 (0.1%)	25.0
Cupric carbonate	10.5	Vitamin A palmitate (500000 IU/g)	0.0
Potassium iodate	0.35	Vitamin D3 (400000 IU/g)	2.5
Sodium selenate	0.36	Vitamin K1/Dextrose mix (10 mg/g)	75.0
Ammonium paramolybdate·$4H_2O$	0.28		
Sodium metasilicate·$9H_2O$	50.75		
Chromium potassium sulfate·$12H_2O$	9.63		
lithium chloride	0.61		
Boric acid	2.85		
Sodium fluoride	2.22		
Nickel carbonate	1.11		
Ammonium vanadate	0.23		

[a.] Sucrose powder is used as carrier in the premix and makes the remaining amount of premix in the diet.

Table S3. The primer sequences

Gene symbol	Gene name	Primer	Sequence (5'→3')
ALOX5	Arachidonate 5-lipoxygenase	F	CATCTGCTGGACAAGGCATTC
		R	GTGGGGTGTCTTCAGTGTGAT
ALOX8	Arachidonate 8-lipoxygenase	F	TCAGTTCAGGCCAGTTCGAC
		R	GGGAGCGTGGCTATGAAACT
COL1A1	Collagen, type I, alpha 1	F	CGATGGATTCCCGTTCGAGT
		R	GAGGCCTCGGTGGACATTAG
CRP	C-reactive protein	F	GTCTGCTACGGGGATTGTAGA
		R	CACCGCCATACGAGTCCTG
CYP2J9	Cytochrome P450 2J9	F	CAACCTAACTGCACTGCACAG
		R	GGCTTTCTACTTACTGAACCC
EPHX2	Epoxide hydrolase 2	F	CTGTGGCCAGTTTGAACACG
		R	ATCACTGGCTCGGAAGAAGC
GAPDH	Glyceraldehyde-3-phosphate dehydrogenase	F	GCATCTTCTTGTGCAGTGCC
		R	TACGGCCAAATCCGTTCACA
GPX4	Glutathione peroxidase 4	F	AGTACAGGGGTTTCGTGTGC
		R	CATGCAGATCGACTAGCTGA
IL6	Interleukin 6	F	TACCACTTCACAAGTCGGAGG
		R	CTGCAAGTGCATCATCGTTGTTC
LPCAT3	Lysophosphatidylcholine acyltransferase 3	F	AGATGGAATTCCTCATTGTTATCGT
		R	GAAGGGCTGTAGGGCAGTGA
PLA2G4A	Phospholipase A2 group IVA	F	CAGCACATTATAGTGGAACACCA
		R	AGTGTCCAGCATATCGCCAAA
PLA2G6	Phospholipase A2 Group VI	F	AGATGTCTTTCGTCCCAGCA
		R	ATGATGTCGGACCCTAGCTG
PNPLA8	Patatin-like phospholipase domain containing 8	F	TCGGTGGTTACATTGGTGGA
		R	GCTGAAGTAGCACACGCTTT
PTGS2	Prostaglandin-endoperoxide synthase 2	F	GCTCAGCCAGGCAGCAAATC

		R	CACCATAGAATCCAGTCCGGG
STRA6	Stimulated by retinoic acid 6	F	TGAGGATCCTAAGATCTACAAGCA
		R	TCAGGAATCCAAGACCCAGA
TGFB1	Transforming growth factor, beta 1	F	CTGCTGACCCCCACTGATAC
		R	AGCCCTGTATTCCGTCTCCT
TNF	Tumor necrosis factor	F	CCTGTAGCCCACGTCGTAG
		R	GGGAGTAGACAAGGTACAACCC

Table S4. Relative change (Log_2 fold change) and FDR-adjusted p-values of all oxylipins detected in liver. N=10 mice per treatment was utilized for this analysis.

**Hepatic oxylipin**	**Log_2(Fold change): VD/VR**	**FDR-adjusted p-value**
5,6-DiHETrE	2.17	< 0.001
19,20-DiHDPE	1.66	< 0.001
16,17-DiHDPE	1.52	< 0.001
8,9-DiHETrE	1.47	< 0.001
11,12-DiHETrE	1.47	< 0.001
20-HETE	2.69	< 0.001
10(11)-EpDPE	2.03	< 0.001
13,14-DiHDPE	1.46	< 0.001
14,15-DiHETrE	1.33	0.001
10,11-DiHDPE	1.80	0.002
9,10-DiHOME	1.23	0.002
11(12)-EpETrE	4.52	0.003
12-HETE	1.82	0.005
12(13)-EpOME	3.44	0.010
17,18-DiHETE	2.09	0.011
5-HETE	1.01	0.011
9(10)-EpOME	3.34	0.013
8(9)-EpETrE	4.20	0.017
12,13-DiHOME	0.82	0.020
15-oxo-ETE	2.24	0.027
LTB_4	1.56	0.027
14(15)-EpETrE	3.79	0.045
9-HETE	0.49	0.045
11-HETE	0.54	0.050
15-deoxy-PGJ2	-0.62	0.070
EKODE	1.59	0.119
11,12,15-TriHETrE	1.19	0.119
9,10,13-TriHOME	1.70	0.133
13-HODE	-0.67	0.133
9,12,13-TriHOME	1.39	0.160
8-HETE	1.46	0.177
15,16-DiHODE	1.16	0.187
9-oxo-ODE	1.30	0.215
TXB2	-1.46	0.263
14,15-DiHETE	0.88	0.269
9-HODE	-0.45	0.353
15(S)-HETrE	-0.16	0.797
15-HETE	-0.03	0.797
6-keto-PGF1a	-0.29	0.818

Table S5. Relative change (Log_2 fold change) and FDR-adjusted p-values of all oxylipins detected in cerebrum. N=10 mice per treatment was utilized for this analysis.

Cerebral oxylipin	Log_2(Fold change): VD/VR	FDR-adjusted p-value
5-oxo-ETE	-2.47	0.003
4,5-DiHDPE	-2.30	0.012
9,10-DiHOME	-1.99	0.012
5-HETE	-1.89	0.012
12,13-DiHOME	-1.78	0.012
16(17)-EpDPE	1.68	0.012
11(12)-EpETE	1.59	0.012
15,16-DiHODE	-1.44	0.016
10(11)-EpDPE	1.50	0.016
14,15-DiHETE	-1.48	0.016
5,6-DiHETE	-1.48	0.021
14,15-DiHETrE	-2.09	0.021
8,9-DiHETrE	-1.74	0.021
8-HEPE	1.74	0.021
6-keto-PGF1a	-1.56	0.021
16,17-DiHDPE	-1.44	0.021
15-HETE	-1.34	0.021
5-HEPE	-1.26	0.021
19,20-DiHDPE	-2.32	0.022
10,11-DiHDPE	-1.67	0.022
17(18)-EpETE	1.42	0.022
5,15-DiHETE	-1.09	0.022
8-HETE	-1.01	0.022
15(S)-HETrE	-1.19	0.025
12,13-DiHODE	-4.05	0.026
13,14-DiHDPE	-1.62	0.026
13(14)-EpDPE	1.20	0.028
11,12-DiHETrE	-1.62	0.031
7,8-DiHDPE	-1.90	0.034
THF diol	-1.29	0.034
5,6-DiHETrE	-1.55	0.035
15-oxo-ETE	1.30	0.035
9-HETE	-1.07	0.035
TXB2	-1.35	0.043
5(6)-EpETrE	-3.47	0.058
17,18-DiHETE	-1.41	0.058
11,12-DiHETE	-1.02	0.059
19(20)-EpDPE	-0.88	0.059
9-oxo-ODE	-1.43	0.061
PGE2	-0.79	0.066
8,9-DiHETE	-0.79	0.079
9-HOTrE	-1.21	0.080

PGF2a	-0.91	0.080
11-HETE	-0.53	0.087
9,10,13-TriHOME	-0.91	0.106
8(9)-EpETE	0.93	0.107
9-HODE	-0.43	0.107
PGE3	-0.88	0.108
9,12,13-TriHOME	-0.75	0.108
PGD1	-0.53	0.108
8,15-DiHETE	-1.00	0.112
11,12-,15-TriHETrE	-0.68	0.114
20-HETE	-1.17	0.123
EKODE	-0.92	0.134
13-HODE	0.21	0.134
12(13)-EpOME	-0.72	0.145
LTB3	-0.59	0.149
PGE1	-0.42	0.157
11(12)-EpETrE	-0.76	0.166
6-trans-LTB4	-0.64	0.166
8(9)-EpETrE	1.12	0.175
13-HOTrE	-0.28	0.185
9(10)-EpOME	-0.66	0.206
LTB4	-0.30	0.213
PGD2	-0.71	0.243
12-HETE	-1.03	0.338
15-deoxy-PGJ2	-0.20	0.353
20-COOH-LTB4	-0.85	0.438
PGD3	-0.41	0.516
7(8)-EpDPE	-0.29	0.521
13-oxo-ODE	-0.03	0.521
PGJ2	-0.07	0.536
15(16)-EpODE	-0.83	0.575
9,10-DiHODE	-0.69	0.613
12(13)-EpODE	0.64	0.962
PGB2	0.28	0.982
9(10)-EpODE	0.70	0.997

Table S6. Relative change (Log$_2$ fold change) and FDR-adjusted p-values of all oxylipins detected in plasma.

Plasma oxylipin	Log$_2$(Fold change): VD/VR	FDR-adjusted p-value
11(12)-EpETE	-0.59	0.65
TXB2	-0.57	0.65
9(10)-EpODE	-0.55	0.65
16(17)-EpDPE	-0.52	0.65
13-HODE	-0.46	0.65
9-HOTrE	-0.31	0.65
15-oxo-ETE	-0.18	0.65
9-oxo-ODE	-0.08	0.65
11,12,15-TriHETrE	0.90	0.69
15,16-DiHODE	0.61	0.69
13(14)-EpDPE	-0.61	0.69
EKODE	0.40	0.69
6-trans-LTB4	0.31	0.69
10(11)-EpDPE	-0.29	0.69
14(15)-EpETrE	-0.23	0.69
9-HODE	-0.16	0.69
12(13)-EpOME	0.08	0.69
8(9)-EpETrE	-0.08	0.69
11(12)-EpETrE	-0.05	0.69
9(10)-EpOME	0.05	0.69
8-HETE	0.02	0.69
12-HETE	0.54	0.70
14,15-DiHETrE	-0.25	0.70
9,12,13-TriHOME	0.16	0.70
11,12-DiHETrE	-0.19	0.71
15-HETE	0.09	0.71
5,6-DiHETrE	0.28	0.74
9,10,13-TriHOME	0.19	0.74
13-HOTE	0.20	0.75
15(16)-EpODE	-0.16	0.75
11-HETE	0.09	0.75
9-HETE	0.13	0.84
5-HETE	0.06	0.85
8,9-DiHETrE	0.08	0.89
9,10-DiHOME	0.04	0.89
12,13-DiHOME	0.04	0.89
15-deoxy-PGJ2	0.17	0.98
20-COOH-LTB4	0.99	0.99
